# Effects of dietary xylanase supplementation and insoluble fiber on growth performance, cecal microbial population and intestinal histomorphology in broiler chickens fed wheat-based diet

**DOI:** 10.1016/j.psj.2025.105749

**Published:** 2025-08-28

**Authors:** Masoud Karimipour, Somayyeh Salari, Ali Aghaei

**Affiliations:** Department of Animal Science, Animal Science and Food Technology Faculty, Agricultural Sciences and Natural Resources University of Khuzestan, Ahvaz, P.O. Box: 6341773637, Iran

**Keywords:** Xylanase supplement, Viscosity, Histomorphometry, Insoluble fiber

## Abstract

This study was conducted to evaluate the effect of dietary xylanase supplementation and insoluble fiber on growth performance, microbial populations, and histomorphology of broiler chickens fed wheat-based diets. A total of 300 one-day-old male chicks were assigned in completely randomized design with a factorial arrangement 2 × 3 using 6 treatments and five replicates (10 chicks per replicate) from 1 to 42 days of age. Treatments were insoluble fiber source (none (CTL), 3 % wheat bran (WB), and 3 % rice bran (RB)) and xylanase supplementation (with (+) and without (_) in a wheat-based diets. Dietary supplementation of xylanase with insoluble fiber resulted in a reduction of overall feed intake (FI). The body weight gain (BWG) of birds fed the CTL diet in the absence of enzyme increased compared to the enzyme-containing diet, but the opposite effect was observed at the presence of WB. The feed conversion ratio (FCR) was markedly decreased by xylanase supplementation, both independently and in combination with insoluble fiber, during the finisher phase and the entire period of experiment. Dietary supplementation of xylanase at the presence of WB and RB increased the cecal microbial population of *Lactobacillus* and *coliforms*. The duodenal pH decreased by supplementation of xylanase in CTL and RB diet. The addition of xylanase in CTL or insoluble fiber diet decreased ileal viscosity, however, it did not have effect on litter moisture. Dietary supplementation of xylanase in CTL and WB diet increased the villus height of jejunum .Supplementation of xylanase to diets containing WB and RB increased the villus height to crypt depth ratio in the jejunum. It can be concluded that xylanase supplementation in diets containing insoluble fiber, positively influenced growth performance, cecal microbial composition, and intestinal histomorphometry in broiler chickens.

## Introduction

The use of wheat and barley in broiler chicken nutrition have notable adverse effects on feed efficiency and growth (Slominski, 2011). Some anti-nutritional factors present in these grains are non-starch polysaccharides (NSP). NSP are responsible for a slow rate of gastro-intestinal passage in broiler chickens. This process lowers the activity of digestive enzymes and makes the feed less digestible (Shihab et al., 2025). Also, NSP can make the gut more viscous and provide food for microbiota and pathogenic bacteria like *Clostridium perfringens* (Kaldhusdal and Lovland, 2000). Endo-β−1,4-xylanases are key enzymes in the degradation of arabinoxylans, the main NSP in wheat. In vivo production of arabinoxylan oligosaccharides can have an effect on the microbiota in the gut. These compounds can improve gut integrity, improve the function of the gut epithelial barrier, and activate the intestinal lymphatic system (Bedford, 2000). Furthermore, research shows that adding insoluble fiber sources to high-viscosity diets may be beneficial for the nutrition of broilers (Pourazadi et al., 2020b). Wheat bran (**WB**) is a source of insoluble fiber, rich in arabinoxylan and cellulose (Kim et al., 2006). Previous study have shown that WB improved microbial composition (Shang et al., 2020). There was also evidence showing beneficial effects of WB-derived arabinoxylan on overall poultry health (Veluri et al., 2024). Adding 3 % WB to the diets of broilers has been shown to make it easier for them to digest protein and energy, increase the villus height of jejunum and ileum, and the activity of digestive enzymes and antioxidants (Shang et al., 2020). Rice bran (**RB**) is an additional source of insoluble fiber and can be a cost-effective feed ingredient for poultry. It is a valuable feed ingredient rich in amino acids, starch, fat, vitamins and some trace minerals (Qi et al., 2015). Arabinoxylan is the main polysaccharide in the fiber fraction of rice (Mendez-Encinas et al., 2018). Supplementation of xylanase to the diet containing arabinoxylan can make stimbiotic (**STB**). Stimbiotic is able to accelerate fiber-degrading microbiota establishment in GIT to maximum the utilization of dietary fiber, especially arabinoxylans, which is the mayor component of dietary fiber in practical diets (González-Ortiz et al., 2019). It has been recently reported that STB could improves broilers performance in an experimentally induced necrotic enteritis infection (J. H. Lee et al., 2022). Also, STB has been shown to improve nutrient digestibility, increase beneficial bacteria, and enhance broiler performance (Song et al., 2022). Therefore, there is a clear need to further investigate the combined effects of wheat bran and rice bran, in the presence of xylanase, on broiler nutrition and intestinal health. While previous studies have separately highlighted the benefits of WB, RB, and stimbiotics, their potential synergistic impact when included together in wheat-based diets has not been fully explored. The present study was therefore conducted to address this gap. It was hypothesized that dietary supplementation with WB and RB, in combination with xylanase, would exert synergistic effects leading to improved growth performance, nutrient digestibility, and intestinal health in broiler chickens fed wheat-based diets.

## Materials and methods

### Chemical analysis of insoluble fiber

Wheat, WB, and RB samples were randomly obtained and analyzed using a DA 7250 NIR analyzer (Perten Instruments, Hägersten, Sweden) (Table 1).

**Table 1:** Chemical composition of feedstuffs determined by NIR.

Chemical Composition (%)	RB	WB	Wheat
Moisture	9.77	9.3	7.2
CP	5.58	17.12	9.8
CF	31.25	11.23	-
Ash	4.51	4.6	-
EE	2.89	3.77	-
Starch	-	21.33	3.12
NDF	45.08	-	17.8
ADF	25.72	-	-

Abbreviations: NIR: Near-Infrared Spectroscopy, RB: Rice Bran, WB: Wheat Bran, CP: Crude Protein, CF: Crude Fiber, EE: Ether Extract, NDF: Neutral Detergent Fiber, ADF: Acid Detergent Fiber.

### Birds, diets, and management

The study’s procedures were approved by the Animal Ethics Committee at the Agricultural Sciences and Natural Resources University of Khuzestan, Ahvaz, Iran (Approval no 1864581). A total of 300 one-day-old male broiler chicks (Ross 308) with an initial body weight of 42 ± 2 g were obtained from a commercial hatchery and distributed into 30 floor pens (6 treatments with 5 replicates of 10 birds each, 10 birds per m² in accordance with recommended welfare guidelines) to ensure uniform average bird weight in completely randomised design with factorial arrangement 3 × 2. The dietary treatments comprised insoluble fiber (without fiber (control, **CTL**), 3 % WB, and 3 % RB) and xylanase supplementation (+, -). The xylanase enzyme, isolated from *Aspergillus Niger*, demonstrated an enzymatic activity of 50,000 IU/g (BONALYZE, Bioluence, Tehran, Iran) and was added at 200 gram per ton of the wheat-based diet. Experimental diets were administered in mash form and designed to fulfill the dietary needs of broilers according to Ross 308 (2021) (Table 2). Chicks were raised for 42 days on floor pens. Clean wood shavings were utilized as bedding material. Each pen contained one suspended drinker and one feeder. Feed and water were offered *ad libitum* throughout the experimental period. The continuous lighting schedule was done during the first 3 d after the hatching and for the rest of the experiment, the lighting schedule was changed to 23L: 1D. During the first week, the ambient temperature was 33°C and progressively decreased by 2°C every week with relative humidity kept between 50 and 60 %. Vaccination of broiler chickens against infectious bronchitis and Newcastle disease was carried out on days 2 and 7 by eye-drop and injection, respectively. In addition, on day 15, vaccination against Gumboro disease was administered via drinking water. No therapeutic antibiotics were administered during the experimental period. Coccidiostats were not included in the diet.

**Table 2:** Composition of experimental diets used for feeding broiler chickens (percentage).

Periods	Starter (d 1 to 10)				Grower (d 11 to 24)			Finisher (d 25 to 42)		
Dietary Items	CTL	WB	RB		CTL	WB	RB	CTL	WB	RB
Corn	3.17	0.00	0.00		5.96	2.46	1.18	9.25	7.00	6.69
Wheat	60.00	60.00	60.00		60.00	60.00	60.00	60.00	60.00	60.00
Soybean Meal (480 g CP/kg)	28.00	27.18	27.00		25.00	25.30	26.00	23.00	22.00	23.00
Corn Gluten Meal (540 g CP/kg)	3.47	3.87	4.04		3.50	2.50	3.00	1.50	1.60	1.44
Vegetable Oil	0.20	0.80	0.80		1.00	2.00	2.30	2.00	2.21	1.72
Wheat Bran	0.00	3.00	3.00		0.00	3.00	3.00	0.00	3.00	3.00
L-Methionine	0.38	0.38	0.38		0.30	0.31	0.31	0.28	0.28	0.28
L-Lysine HCl	0.47	0.48	0.50		0.39	0.38	0.37	0.32	0.34	0.32
L-Threonine	0.24	0.25	0.25		0.18	0.20	0.20	0.17	0.17	0.17
Dicalcium Phosphate	2.22	2.19	2.18		1.94	1.90	1.89	1.70	1.67	1.66
Calcium Carbonate	0.72	0.73	0.73		0.66	0.68	0.68	0.60	0.63	0.62
Salt	0.04	0.04	0.04		0.02	0.02	0.02	0.08	0.08	0.08
Sodium Bicarbonate	0.59	0.58	0.58		0.55	0.55	0.55	0.60	0.52	0.52
Vitamin Supplement¹	0.25	0.25	0.25		0.25	0.25	0.25	0.25	0.25	0.25
Mineral Supplement²	0.25	0.25	0.25		0.25	0.25	0.25	0.25	0.25	0.25
Calculated analysis (Percantage, unless stated otherwise)										
AME(kcal/kg)	2931	2917		2920	3016	3011	3021	3093	3062	3026
Crude Protein	22.45	22.56		22.25	21.04	21.03	21.09	19.15	19.09	19.11
Digestible Lysine	1.28	1.28		1.28	1.15	1.15	1.15	1.03	1.03	1.03
Digestible Methionine + Cystine	0.98	0.98		0.98	0.88	0.88	0.88	0.80	0.80	0.80
Digestible Threonine	0.89	0.89		0.89	0.79	0.80	0.81	0.72	0.72	0.72
Digestible Tryptophan	0.24	0.24		0.23	0.22	0.23	0.23	0.21	0.21	0.21
Digestible Arginine	1.21	1.21		1.19	1.13	1.14	1.15	1.04	1.04	1.04
Digestible Isoleucine	0.80	0.80		0.79	0.75	0.74	0.75	0.68	0.67	0.68
Digestible Valine	0.88	0.88		0.86	0.83	0.82	0.83	0.75	0.75	0.75
Calcium	0.96	0.96		0.96	0.87	0.87	0.87	0.79	0.79	0.79
Available Phosphorus	0.48	0.48		0.48	0.43	0.43	0.43	0.39	0.39	0.39

Abbreviations: CTL: Control (without insoluble fiber supplementation), WB: Wheat Bran, RB: Rice Bran.

1 The vitamin supplement provided the following (per kg of diet): Vitamin A, 3,600,000 IU; Vitamin D_3_, 800,000 IU; Vitamin E, 720 IU; Vitamin K, 800 IU; Vitamin B_12_, 6 mg; Thiamine, 709 mg; Riboflavin, 2640 mg; Niacin, 3920 mg; Pyridoxine, 1182 mg; Biotin, 40 mg; Folic Acid, 400 mg; Pantothenic Acid, 11880 mg; Ethoxyquin, 400 mg.

2 The mineral supplement provided the following (per kg of diet): Iron, 30000 mg; Zinc Sulfate, 25870 mg; Manganese Sulfate, 29760 mg; Iodine, 342 mg; Copper Sulfate, 2400 mg; Selenium, 80 mg. Note: The enzyme xylanase was added to the diet at a level of 200 g per ton as a top dressing.

### Growth performance

Body weight gain (BWG) and feed intake (FI) were recorded weekly for each pen. Feed conversion ratio (FCR) was determined by dividing FI by BWG for each experimental phase (starter: 1 to 10 d, grower: 11 to 24 d and finisher: 25 to 42 d) as well as for the total 42-day period (Kasaeizadeh et al., 2025). Mortality remained low (less than 2 %), with no significant differences observed between the groups.

### Size of different organs

At the end of the investigation (42 d of age), two birds from each group were randomly chosen and killed by cervical dislocation and the weight (relative to pre-slaughter body weight) of the breast, thigh, gastrointestinal tract, gizzard, proventriculus, pancreas, liver, and abdominal fat were evaluated (Macambira et al., 2025).

### Cecal microbial population

To assess cecal microbiota, on day 28, the ceca from two birds per replicate were collected. Approximately 1 gram of cecal digesta was aseptically transferred into 9 mL of sterile saline (0.9 % NaCl), and diluted from 10 ^to 1^ to 10^-7^. *Lactobacilli, Coliforms*, and *E. coli* were grown on De Man-Rogosa-Sharpe (**MRS**) agar, Mac Conkey Agar, and Eosin Methylene Blue (**EMB**) Agar, respectively. Plates for *Lactobacillus* have incubated anaerobically for 48 h at 37°C. Microbial populations for *E. coli* and *Coliforms* were counted after aerobic incubation at 37°C for 24 h. All samples were plated in duplicate (Azizi et al., 2025).

### pH of different parts of the digestive tract and ileal viscosity

On d 28, two birds were randomly selected from each pen, weighed, and euthanised by cervical dislocation. Then, the contents of different parts of the GIT (duodenum, jejunum, ileum, and caecum) were mixed with distilled water in a 1:9 ratio and the pH of the contents was measured using a digital pH metre (model 3110 SET2, Germany). To determine digesta viscosity, at 28 d of age, an additional two birds per replicate were randomly chosen and euthanized via cervical dislocation. The small intestine was carefully removed, and ileal digesta (from the region between Meckel’s diverticulum and the ileo-cecal junction) were immediately collected. Samples were placed into sterile tubes and centrifuged at 4,000 × *g* for 10 min at 4°C. The resulting supernatant was used to determine viscosity (centipoise) (**cP**) using a cannon-fenske viscometer (Lázaro et al., 2003).

### Intestinal morphology

On d 42, two birds from each replicate selected to examine morphological alterations of small intestine. The gastrointestinal tract of the chosen birds was excised, with approximately two-centimeter segments extracted from the duodenum, jejunum, and ileum. The collected samples were placed in plastic containers containing a formalin solution10 %. After that, a histokinetics device (Leica, TP102, Germany) was used to make paraffin tissue molds. A microtome (Leitz, Leica, model1512, Germany) was used to cut the tissue into 5–6 micrometer sections, which were then put on microscope slides. These sections were stained utilizing hematoxylin and eosin staining techniques in accordance with standard scientific protocols (Suvarna et al., 2018). An optical microscope (Dino-Capture model AM, made in Germany) and the Dino Capture Software II were used to look at and analyze tissue samples (Liu et al., 2022). The ratio of villus height to crypt depth (**VH/CD**) was calculated subsequently.

### Statistical analysis

All data were analysed as a completely randomised design with six treatments using the GLM technique of SAS software version 9.1 (Sas Institute, 2001). The main effects (insoluble fibre and enzyme supplementation) and interactions were studied in 3 × 2 factorial arrangement. The replication pen was designated as the experimental unit for growth performance parameters, while the individual birds served as the experimental units for the other measurements. Before analysis, normality of all measured parameters was assessed. Viscosity and microbiological counts were subjected to base-10 logarithm transformation and body organ weight, expressed as a proportion, was arcsine-square root transformed before analysis. Significant differences among the treatments were determined using duncan's multiple range test at *P* ≤ 0.05. All data were checked for normality and homogeneity of residuals prior to ANOVA.

The statistical model used was: Y_ijk_​=μ+A_i_​+β_j_​+Aβ_ij _+e_ijk​._ The model incorporated main effects for insoluble fiber (A_i_) and Enzyme (β_j_), as well as an interaction term (Aβ_ij _), with the observed value represented by Y_ijk_, the overall mean by μ, and the random error by e_ijk.._

## Results

### Growth performance

The results of growth performance of broiler chickens during the different period of experiment were shown in Table 3. Significant interactions (*P* ≤ 0.05) between insoluble fiber source and enzyme supplementation were observed for FI, and BWG during all periods of experiment. Supplementation of xylanase to diets containing WB reduced FI throughout the experiment, with a reduction of 3.5 % in the starter, 10.7 % in the grower, and 8.9 % in the finisher period, leading to a 9.0 % decrease in the total experimental period compared with the WB diet without enzyme. In contrast, xylanase supplementation to the CTL diet increased FI by 3.5 % in the starter, 9.0 % in the grower, and 8.3 % in the finisher period, resulting in an overall 8.1 % increase in FI compared with CTL without enzyme. Also, xylanase addition to the CTL diet improved BWG by 13.3 % (starter), 16.3 % (grower), and 27.1 % (finisher), corresponding to a 22.8 % increase overall. However, in the WB diet, enzyme supplementation slightly decreased BWG (0.6 % in starter, 1.4 % in grower, and 4.4 % in finisher), resulting in a 3.4 % reduction over the total period compared with WB without enzyme. No significant BWG differences were observed between RB diets with or without enzyme during the starter and grower phases, though a minor increase of 0.04 % in the finisher and 1.1 % overall was noted.

**Table 3:** Effect of different sources of insoluble fiber and xylanase supplementation on growth performance of broiler chickens fed a wheat based diet.

Periods		Starter (d 1 to 10)			Grower (d 11 to 24)			Finisher (d 25 to 42)			Total period (d 1 to 42)		
Fiber Source	Enzyme	FI	BWG	FCR	FI	BWG	FCR	FI	BWG	FCR	FI	BWG	FCR
CTL	_	248.16^c^	169.83^b^	1.46	752.63^c^	475.45^c^	1.58	2431.58^c^	1117.37^d^	2.17^a^	3432.38^c^	1762.65^d^	1.94^a^
	+	256.96^b^	192.33^a^	1.33	820.57^b^	552.80^a^	1.48	2633.00^b^	1420.09^b^	1.85b^c^	3710.54^b^	2165.22^a^	1.71^c^
WB	_	255.58^b^	189.26^a^	1.35	914.07^a^	529.00^a^	1.72	2806.80^a^	1472.09^a^	1.91b^c^	3976.46^a^	2190.36^a^	1.81^b^
	+	246.58^c^	188.08^a^	1.31	816.07^b^	521.80^a^^b^	1.57	2558.19^b^^c^	1406.81^b^	1.82^c^	3620.84^b^	2115.71^b^	1.71^c^
RB	_	271.93^a^	178.05^b^	1.53	822.92^b^	469.82^c^	1.75	2574.00^b^^c^	1291.86^c^	1.99b^c^	3668.85^b^	1939.72^c^	1.89^a^^b^
	+	255.08^b^	176.55^b^	1.44	785.82b^c^	491.82^c^^b^	1.6	2504.99^b^^c^	1292.35^c^	1.94^c^	3545.87^b^^c^	1960.72^c^	1.80^b^
*SEM*		2.32	2.89	0.02	12.82	10.62	0.03	54.97	13.47	0.04	56.09	14.01	0.03
*Effect of Enzyme*													
_		258.56^a^	179.04^b^	1.44^a^	829.87^a^	491.42^b^	1.68^a^	2604.13	1293.77^b^	2.02^a^	3692.56	1964.24^b^	1.88^a^
+		252.87^b^	185.32^a^	1.36^b^	807.48^b^	522.13^a^	1.55^b^	2565.39	1373.09^a^	1.87^b^	3625.75	2080.55^a^	1.74^b^
*SEM*		1.34	1.67	0.01	7.4	6.13	0.02	31.74	7.77	0.03	32.38	8.09	0.02
*Effect of Insoluble Fiber*													
CTL		252.56^b^	181.08^b^	1.40^b^	786.60^b^	514.13^a^	1.53^b^	2532.29^b^	1268.73^b^	2.01^a^	3571.46^b^	1963.94^b^	1.83^a^
WB		251.08^b^	188.18^a^	1.33^c^	865.07^a^	525.40^a^	1.64^a^	2682.49^a^	1439.45^a^	1.86^b^	3798.65^a^	2153.04^a^	1.76^b^
RB		263.50^a^	177.30^b^	1.48^a^	804.36^b^	480.82^b^	1.67^a^	2539.50^b^	1299.11^b^	1.96^a^	3607.36^b^	1950.22^b^	1.85^a^
*SEM*		1.64	2.04	0.01	9.06	7.51	0.02	38.78	9.52	0.03	39.66	9.91	0.02
*P-value*													
Insoluble Fiber		0.0001	0.003	0.0001	0.0001	0.0009	0.0001	0.01	0.0001	0.0009	0.0009	0.0001	0.02
Enzyme		0.006	0.01	0.0001	0.04	0.001	0.0001	0.39	0.0001	0.0004	0.15	0.0001	0.0001
Insoluble fiber*Enzyme		0.0001	0.003	0.09	0.0001	0.0002	0.55	0.001	0.0001	0.01	0.0001	0.0001	0.04

Abbreviations: FI: Feed Intake, BWG: Body Weight Gain, FCR: Feed Conversion Ratio, CTL: Control diet (basal diet), WB: Wheat bran, RB: Rice bran, _: Without enzyme, +: With enzyme.

a-c Means with different letters within the same column differ significantly (*P* ≤ 0.05).

Significant interactions between insoluble fiber source and enzyme supplementation were observed for FCR at finisher period (*P* = 0.01) and whole period of experiment (*P* = 0.04). Xylanase supplementation markedly improved FCR, particularly in the CTL and WB diets. In the CTL group, FCR decreased by 8.9 % (starter), 6.3 % (grower), and 14.7 % (finisher), with an overall 11.9 % improvement compared to the control diet without enzyme. Similarly, in the WB group, enzyme inclusion reduced FCR by 3.0 % (starter), 8.7 % (grower), and 4.7 % (finisher), leading to a 5.5 % overall improvement. No significant FCR change was observed in RB diets.

### Carcass and digestive trait characteristics

Effects of dietary treatments on carcass and digestive traits of broiler chickens at d 42 represented in Table 4. The interaction between insoluble fiber source and xylanase supplementation was significant for the relative weight of gizzard (*P* = 0.01), proventriculus (*P* = 0.003), duodenum (*P* = 0.001), jejunum (*P* = 0.0009), and ileum (*P* = 0.0001). Enzyme supplementation to the WB diet decreased gizzard weight by 15.7 % compared with WB without enzyme. In the CTL diet, proventriculus weight decreased by 22.9 % with enzyme supplementation. Liver weight decreased by 11.4 % in enzyme-supplemented diets compared with the unsupplemented control. Conversely, enzyme addition reduced duodenum weight in CTL by 22.1 %, jejunum weight by 18.6 %, and ileum weight by 22.9 %.

**Table 4:** Effect of different sources of insoluble fiber and xylanase supplementation on carcass components and internal organs of broiler chickens fed wheat based diet (percentage of live body weight).

Fiber Source	Enzyme	Breast	Thigh	Liver	Gizzard	Proventriculus	Abdominal Fat	Pancreas	Duodenum	Jejunum	ileum	Carcass
CTL	_	23.82	18.87	2.08	2.98^b^^c^	0.48^a^	1.56	0.23	2.31^a^	4.84^a^	4.83^a^	63.85
	+	25.39	18.77	2.36	3.42^a^^b^	0.37^c^^b^	1.9	0.19	1.80^b^	3.94^b^	3.72^c^	63.67
WB	_	24.32	19.14	2.02	3.25^a^^b^	0.41^a^^b^^c^	1.92	0.2	1.80^b^	3.80^b^	3.74^c^	64.76
	+	23.96	19.97	2.18	2.74^c^	0.42^a^^b^^c^	1.95	0.21	1.87^b^	3.94^b^	4.00^b^^c^	64.92
RB	_	25.48	18.27	2.17	3.46^a^	0.35^c^	1.6	0.25	1.87^b^	3.93^b^	3.92^b^^c^	63.7
	+	25.54	18.88	2.59	3.24^a^^b^	0.43^a^^b^	1.71	0.24	2.01^b^	3.24^b^	4.24^b^	66.42
*SEM*	0.88	0.49	0.13	0.14	0.01	0.1	0.01	0.08	0.17	0.17	0.9	
*Effect of Enzyme*												
_		24.96	18.76	2.38^a^	3.23	0.41	1.85	0.23	1.99	4.2	4.16	65
+		24.54	19.21	2.09^b^	3.13	0.41	1.69	0.21	1.89	4.99	3.99	64.1
*SEM*		0.51	0.28	0.08	0.08	0.0001	0.06	0.01	0.04	0.1	0.1	0.52
*Effect of Insoluble Fiber*												
CTL		24.61	18.27	2.22	3.20	0.43	1.73^a^	0.21^b^	2.06^a^	4.43^a^	4.28^a^	65.06
WB		24.14	19.56	2.1	3.00	0.41	1.94^a^^b^	0.20^b^	1.84^b^	3.87^b^	3.87^b^	64.84
RB		25.51	18.58	2.38	3.35	0.39	1.66^b^	0.24^a^	1.94^a^^b^	4.08^a^^b^	4.08^a^^b^	63.76
*SEM*		0.62	0.34	0.09	0.1	0.00001	0.07	0.01	0.05	0.12	0.12	0.64
*P-value*												
Insoluble Fiber		0.3	0.13	0.13	0.07	0.41	0.03	0.009	0.04	0.04	0.04	0.31
Enzyme		0.56	0.27	0.01	0.44	0.78	0.06	0.23	0.15	0.15	0.16	0.23
Insoluble fiber*Enzyme		0.52	0.61	0.65	0.01	0.003	0.32	0.24	0.001	0.0009	0.0001	0.21

Abbreviations: CTL: Control diet (basal diet), WB: Wheat bran, RB: Rice bran, _: Without enzyme, +: With enzyme.

a- cMeans with different letters within the same column differ significantly (*P* ≤ 0.05). SEM = Standard Error of the Mean.

### Cecal microbial population, pH of gastrointestinal tract, litter moisture, and ileal viscosity

At 28 days of age, a significant interaction between insoluble fiber source and xylanase supplementation was observed for cecal microbial population of Coliform (*P* = 0.0001) and Lactobacillus (*P* = 0.0001), pH of gastrointestinal tract (*P* = 0.02), litter moisture (*P* = 0.03), and ileal viscosity (*P* = 0.0001) (Table 5). *Coliform* counts increased by 12.9 % in WB with enzyme and 8.2 % in RB with enzyme compared with their respective unsupplemented diets. *Lactobacillus* counts increased by 8.8 % in WB with enzyme and 22.9 % in RB with enzyme. Enzyme supplementation decreased ileal viscosity by 13.9 % in CTL, 4.7 % in WB, and 11.6 % in RB compared with their respective controls. Litter moisture was reduced by 22.3 % in WB with enzyme and 8.4 % in RB with enzyme compared with diets without enzyme, the reduction being more pronounced in WB diets.

**Table 5:** Effects of different sources of insoluble fiber and xylanase supplementation on pH of various sections of the gastrointestinal tract, cecal microbial population (log CFU/g), ileal viscosity (cPs), and litter moisture (%) in broiler chickens fed wheat based diets.

		pH					**Cecal Microbial Population**			**Viscosity and litter Moisture**		
Fiber Source	Enzyme	Gizzard	Duodenum	Jejunum	ileal	Cecum	TBP	*Coliforms*	*Lactobacillus*	*Escherichia*	Viscosity	Moisture
CTL	**_**	2.62	5.38^b^	5.54	6.53	5.5	8.86	8.58^d^	7.95^d^	6.68	2.65^a^	28.68^a^
	**+**	2.19	5.12^c^	5.26	6.53	5.04	9.02	9.18^b^	7.60^e^	6.69	2.28^d^	26.90^a^^b^
WB	**_**	2.70	5.48^b^	5.55	6.54	5.69	9.36	8.27^e^	8.05^c^	6.72	2.35^c^	22.28^c^
	**+**	2.33	5.43^b^	5.39	6.27	5.32	9.73	9.34^a^	8.76^b^	6.73	2.24^e^	26.25^a^^b^^c^
RB	**_**	2.62	5.74^a^	5.43	6.47	5.73	9.27	8.27^e^	7.96^d^	6.74	2.49^b^	26.27^a^^b^^c^
	**+**	2.65	5.25^b^^c^	5.34	6.32	5.32	9.74	8.94^c^	9.78^a^	6.82	2.20^f^	22.85^b^^c^
*SEM*	0.1	0.07	0.09	0.09	0.07	0.1	0.03	0.03	0.11	0.01	1.41	
*Effect of Enzyme*												
_		2.65^a^	5.54^a^	5.51^a^	6.51	5.64^a^	9.16^b^	8.37^b^	7.79^b^	6.74	2.50^a^	25.74
+		2.39^b^	5.27^b^	5.33^b^	6.37	5.23^b^	9.50^a^	9.15^a^	8.71^a^	6.72	2.24^b^	25.33
*SEM*		0.06	0.04	0.05	0.05	0.04	0.06	0.02	0.02	0.004	0.01	0.81
*Effect of Insoluble Fiber*												
CTL		2.40	5.25^b^	5.40	6.53	5.27^b^	8.94^b^	8.88^a^	7.77^c^	6.68	2.47^a^	27.79^a^
WB		2.52	5.46^a^	5.47	6.40	5.50^a^	9.55^a^	8.80^b^	8.40^b^	6.72	2.30^c^	24.69^b^
RB		2.63	5.50^a^	5.39	6.42	5.52^a^	9.50^a^	8.61^c^	8.87^a^	6.78	2.35^b^	24.56^b^
*SEM*		0.07	0.05	0.07	0.06	0.05	0.07	0.02	0.02	0.08	0.01	1.00
*P-value*												
Insoluble Fiber		0.11	0.0006	0.63	0.28	0.003	0.0001	0.0001	0.0001	0.71	0.0001	0.03
Enzyme		0.006	0.0002	0.03	0.06	0.0001	0.0005	0.0001	0.0001	0.85	0.0001	0.72
Insoluble fiber*Enzyme		0.07	0.02	0.64	0.34	0.83	0.31	0.0001	0.0001	0.9	0.0001	0.03

Abbreviations: CTL: Control diet (basal diet), WB: Wheat bran, RB: Rice bran, _: Without enzyme, +: With enzyme, TBP: Total Bacterial Population.

a-e Means with different letters within the same column differ significantly (*P* ≤ 0.05). SEM = Standard Error of the Mean.

### Intestinal morphology

A significant interaction between insoluble fiber source and xylanase supplementation was observed for villus height (VH) in duodenum, jejunum, and ileum (*P* = 0.0001) (Table 6). Xylanase supplementation increased villus height (VH) in all segments of the intestine. In the duodenum, VH increased by 8.8 % in CTL, 1.2 % in WB, and 1.7 % in RB compared with the unsupplemented diets. Villus height to crypt depth ratio (VH:CD) in the jejunum improved by 23.2 % in CTL, 31.4 % in WB, and 32.5 % in RB diets supplemented with enzyme. Crypt depth decreased by 13.4 % in the duodenum and 11.8 % in the jejunum with enzyme supplementation. Additionally, goblet cell numbers increased by 10 % in the duodenum, 11.1 % in the jejunum, and 30 % in the ileum in WB diets supplemented with enzyme.

**Table 6:** Effects of different sources of insoluble fiber and xylanase supplementation on intestinal histomorphometry of small intestine in broiler chickens fed wheat-based diet at 42 days of age.

		Duodenum (μm)				Jejunum (μm)				ileal (μm)			
Fiber Source	Enzyme	VH	CD	GC	VH:CD	VH	CD	GC	VH:CD	VH	CD	GC	VH:CD
**CTL**	_	1127.95^d^	267.90^a^	7.00^d^	4.21^d^	803.43^d^	216.80^a^	10.00^c^	3.70^d^	462.87^d^	143.04^f^	10.00^c^	3.23^d^^e^
	+	1227.34^c^	232.03^c^	9.00^c^	5.29^c^	872.63^b^	191.19^b^	8.00^e^	4.56^c^	640.75^a^	162.71^a^	10.00^c^	3.93^b^
**WB**	_	1235.11^c^	218.17^d^	10.00^b^	5.66^b^	802.45^d^	217.84^a^	9.00^d^	3.68^d^	644.40^a^	156.70^c^	10.00^c^	4.11^a^
	+	1250.22^c^	210.11^e^	11.00^a^	5.95^a^	853.63^c^	126.39^d^	10.00^c^	6.75^a^	482.42^c^	150.91^d^	13.00^a^	3.19^e^
**RB**	_	1367.70^b^	243.50^b^	10.00^b^	5.61^b^	745.46^e^	216.51^a^	12.00^a^	3.44^e^	607.17^b^	170.68^a^	9.00^d^	3.55^c^
	+	1391.26^a^	245.11^b^	10.00^b^	5.67^b^	890.19^a^	146.14^c^	11.00^b^	6.09^b^	487.29^c^	146.83^e^	12.00^b^	3.32^d^
***SEM***	7.93	0.97	0.001	0.03	5.60	1.25	0.001	0.04	3.31	1.07	0.001	0.03	
***Effect of Enzyme***													
**_**		1243.58^b^	243.19^a^	9.00^b^	5.16^a^	783.78^b^	217.05^a^	10.33^a^	3.83^b^	571.48^a^	156.81^a^	9.67^b^	3.63^a^
**+**		1289.60^a^	229.07^b^	10.00^a^	5.63^b^	872.15^a^	154.57^b^	9.67^b^	5.47^a^	536.82^b^	153.48^b^	11.67^a^	3.48^b^
***SEM***		4.57	0.56	0.008	0.02	3.23	0.72	0.009	0.02	1.91	0.62	0.005	0.01
***Effect of insoluble fiber***													
**CTL**		1177.64^c^	249.95^a^	8.00^c^	4.75^c^	838.07	203.99^a^	9.00^c^	4.13^c^	551.81^b^	152.87^b^	10.00^c^	3.58^b^
**WB**		1242.66^b^	214.14^c^	10.50^a^	5.80^a^	828.04	172.11^c^	9.50^b^	5.21^a^	563.41^a^	153.80^b^	11.50^a^	3.65^a^
**RB**		1379.48^a^	244.30^b^	10.00^b^	5.64^b^	817.83	181.32^b^	11.50^a^	4.60^b^	547.23^b^	158.75^a^	10.50^b^	3.44^c^
***SEM***		5.60	0.69	0.001	0.02	3.96	0.88	0.01	0.03	2.34	0.76	0.001	0.02
***P-value***													
**Insoluble Fiber**		0.0001	0.0001	0.0001	0.0001	0.005	0.0001	0.0001	0.0001	0.002	0.0001	0.0001	0.0001
**Enzyme**		0.0001	0.0001	0.0001	0.0001	0.08	0.0001	0.0001	0.0001	0.0001	0.0009	0.0001	0.0001
Insoluble fiber*Enzyme		0.0001	0.0001	0.0001	0.0001	0.0001	0.0001	0.0001	0.0001	0.0001	0.0001	0.0001	0.0001

Abbreviations: CTL: Control diet (basal diet), WB: Wheat bran, RB: Rice bran, _: Without enzyme, +: With enzyme, VH: Villus height, CD: Crypt depth, GC: Goblet cell, VH: CD: Villus height: crypt depth ratio. SEM = Standard Error of the Mean.

a-e Means with different letters within the same column differ significantly (*P* ≤ 0.05).

## Discussion

In general, supplementing xylanase positively influenced FCR in broiler chickens, irrespective of diet. However, insoluble fiber source appeared to dictate the mechanism action of bird performance differently. With the CTL diet, the FCR improvement was closely associated with increased BWG whereas with the WB and RB diets, it was with reduced FI. A possible assumption is that supplementing xylanase to the WB and RB diets may have promoted more fibre fermentation and production of short-chain fatty acids more as an additional source of energy, compared to the CTL group, which could have contributed to improved feed efficiency along with lower FI. This shift in energy harvest from direct absorption to microbial fermentation represents a key metabolic adaptation driven by the enzyme's action on specific fiber types (Kiarie et al., 2013). Similarly, Morgan et al. (2022) observed improved feed efficiency associated with decreased feed intake upon xylanase supplementation in broilers fed diets based on wheat. In the present study, xylanase supplementations markedly reduced the illeal viscosity. This suggests successful hydrolysis of wheat arabinoxylans upon supplementation of xylanase. Furthermore, the improved FCR may be associated with reduced digesta viscosity, indicating depolymerization and the formation of more readily fermentable, medium-chain-length soluble arabinoxylans. The reduction in viscosity is a primary mechanism, as it decreases the diffusion rate of nutrients and digestive enzymes, thereby impairing digestion; xylanase alleviates this physical barrier (Choct, 2015). In this respect, Francesch et al. (2012), reported a reduction in FI when endo -xylanase was included in wheat-based diets. Conversely, Pourazadi et al. (2020b (reported not significant effect on FI when birds fed different sources of insoluble fiber (sunflower hulls (SFH), sugarcane bagasse (SB) or WB) in wheat-based diets. The divergent results on FI across studies could be attributed to differences in the soluble fiber content and initial viscosity of the basal diets, which influences the magnitude of the enzyme's effect (Cowieson et al., 2009). The positive response to insoluble fiber highlights its role in improving gizzard function and enhancing gut motility, which synergizes with xylanase to optimize nutrient utilization (Kiarie et al., 2013). Furthermore, Veluri et al. (2024a), investigated the interactions between stimbiotic supplementation and WB in corn or wheat-based diets, and reported not effect on BWG. Furthermore, Lee et al. (2024), observed that increasing enzyme activity from 1,200 to 240,000 U/g significantly improved BWG throughout all periods of experiment. They also found that a heat-stable xylanase enhanced FCR across all periods, which is in accordance with our findings. The importance of enzyme thermostability is critical, as it ensures the enzyme survives the pelleting process and remains active in the gastrointestinal tract to exert its beneficial effects (Juturu and Wu, 2014). The positive effects of WB and RB with xylanase on BWG and FCR in broilers are likely due to improved nutrient digestibility, reducing viscosity, promoting beneficial bacteria, and improving gut structure.

The results of this experiment showed that xylanase supplementation in WB diet, reduced relative weight of gizzard. This reduction can be logically attributed to the enzymatic breakdown of soluble and insoluble arabinoxylans, which decreases the viscosity and water-holding capacity of the digesta, thereby reducing the mechanical workload and muscular development of the gizzard (Gutierrez et al., 2009). These findings were consistent with those reported by Daymeh et al. (2013), where gizzard weight decreased by the inclusion of enzyme with WB in corn-based diet. The gizzard’s greater fiber retention capacity, especially for insoluble fiber, which leads to prolonged residence time and reduced gizzard weight, contradicts the hypothesis that insoluble fiber accelerates gastrointestinal transit (Hetland et al., 2005). This apparent contradiction underscores the complex role of fiber physico-chemical properties; enzyme supplementation specifically modulates these properties, leading to a less abrasive and more easily processed digesta that requires less muscular effort to grind. Reduced weight of internal organs in broiler chickens following xylanase administration may be attributable to improved digestion, decreased volume of indigestible matter, and altered energy distribution (Moita and Kim, 2022). Furthermore, the metabolic energy typically allocated for the maintenance and function of a hypertrophied gizzard can be redirected towards growth processes, explaining the overall improvement in feed efficiency often associated with enzyme use (Kiarie et al., 2013).

Cecal microbial populations of C*oliforms* and *Lactobacillus* increased in the diet containing WB with enzyme and in the diet containing RB with enzyme, respectively. This selective modulation suggests that different fiber substrates (wheat bran vs. rice bran) release distinct types of oligosaccharides upon enzymatic hydrolysis, which selectively favor the proliferation of specific bacterial genera (Cheng et al., 2021). In this respect, Liu and Kim (2017), reported xylanase supplementation resulted in an augmentation of *Lactobacillus* bacterial populations. Engberg et al. (2004), indicated that xylanase supplementation did not markedly influence the populations of *Coliforms* or *Lactobacillus* bacteria in the cecum, which is in contrast with the findings of the current study. These discrepancies could be attributed to differences in the enzyme preparation's specificity, the composition of the basal diet, or the initial gut microbiota composition of the birds, highlighting the context-dependent nature of xylanase effects (Kers et al., 2018). It has been reported that the augmented microbial population linked to xylanase application may result from the liberation of arabinoxylan of insoluble fiber (Lee et al., 2024; Tiwari et al., 2020). These liberated arabinoxylan-oligosaccharides (AXOS) are not efficiently absorbed in the small intestine and thus serve as fermentable substrates for cecal bacteria, stimulating their growth and metabolic activity Broekaert et al., 2011). Consequently, the use of xylanase supplementation with insoluble fiber sources, such as RB, has demonstrated enhanced prebiotic and stimbiotic benefits. The increased cecal microbial population of *coliforms* in birds fed diets supplemented with WB and xylanase may result from enhanced nutrient availability for their growth following the enzymatic degradation of non-starch polysaccharides (NSPs) (Bimrew, 2014). While an increase in coliforms is often viewed negatively, in this context, it may indicate a general increase in fermentative activity and nutrient turnover, rather than a rise in pathogenic strains, especially if overall bird health and performance are not compromised (Apajalahti et al., 2004).

The xylanase supplementation in all diets caused a decrease in pH in different parts of GIT, which was greater in the CTL and RB diets. This pronounced effect in the low-fiber control (CTL) and rice bran (RB) diets suggests that xylanase not only degrades insoluble fiber but also unlocks fermentable substrates from other diet components, such as soluble non-starch polysaccharides in corn, leading to more uniform and efficient fermentation (Choct, 2015). In contrast, Abdollahi et al. (2021), examined the effects of fiber sources and xylanase supplementation on laying hens fed corn-based diets and reported no impact of enzyme on the pH of internal organs. This divergence from our findings could be due to physiological differences between broilers and laying hens, particularly in feed passage rate and gut morphology, which significantly affect the extent and site of microbial fermentation (Apajalahti et al., 2004).

Furthermore, the decrease in pH observed in internal organs when xylanase supplementation is combined with insoluble fiber may be attributed to alterations in the production of short-chain fatty acids (SCFAs), which, in turn, can influence the pH balance within the internal organs (Tejeda and Kim, 2021). Specifically, the hydrolysis of arabinoxylan chains generates xylo-oligosaccharides (XOS) that are fermented primarily in the lower gut, producing significant quantities of SCFAs like acetate, propionate, and butyrate, thereby acidifying the cecal and colonic environment (Broekaert et al., 2011).

In the present study, xylanase supplementations markedly reduced the digesta viscosity throughout the ileum in CTL, WB, and RB diets. This suggests successful hydrolysis of arabinoxylans of wheat and also WB and RB upon supplementation of xylanase. The significant reduction in the corn-based control (CTL) diet is particularly noteworthy, as it indicates that even low levels of soluble arabinoxylans present in corn and its by-products are sufficient to contribute to digesta viscosity and are effectively targeted by the xylanase (Malathi and Devegowda, 2001). In this respect, Toghyani et al. (2025) reported both xylanase and stimbiotic supplementations reduced the digesta viscosity throughout the small intestine only in wheat-barley fed birds compare to the corn-base diets. The discrepancy with our findings, which showed efficacy in corn-based diets, could be attributed to the specific xylanase used; our enzyme may possess a broader substrate specificity or higher specific activity against the complex arabinoxylan structures found in corn, allowing it to effectively reduce viscosity across diverse dietary backgrounds (Biely et al., 2016). Pourazadi et al. (2024) also reported that in pellet diets, dietary supplementation of SFH plus multi-enzyme carbohydrate resulted in lower ileum viscosity compared to the enzyme, SFH and CTL, whereas, in mash diets, there was no effect. This interaction with feed processing highlights a critical point: the thermal stability of the enzyme during pelleting and its subsequent release in the gut are paramount for its efficacy. The pronounced effect in our study suggests the xylanase used was likely thermostable and survived the pelleting process, allowing it to function effectively in the gastrointestinal tract (Juturu and Wu., 2014).

In the present study, litter moisture was reduced in diets containing WB and RB compare to the CTL group, although this effect was greater in the WB diet without enzymes. This indicates that the physical structure and water-holding capacity of the insoluble fiber itself may play a primary role in improving litter quality by binding moisture within the digesta and reducing excess water excretion, a effect that can be modulated by enzymatic action (Gonzalez-Ortiz et al., 2020). In this respect, Morgan et al. (2022), showed xylanase supplementation in wheat-based diets decreased litter moisture at 35 days of age. The fact that the greatest reduction was observed in the WB diet without enzyme suggests a complex interaction; while xylanase improves nutrient digestibility, it may also break down the fiber matrix that is responsible for its moisture-binding capacity in the hindgut, potentially explaining the attenuated effect when the enzyme was added (Gonzalez-Ortiz et al., 2020). A plausible explanation for the reduced litter moisture content may be attributed to the mitigation of the negative effects of arabinoxylans and a decreased digesta retention time within the gastrointestinal tract (Tiwari et al., 2020; Jiménez-Moreno et al., 2009; Shakouri et al., 2009). Improved nutrient digestibility, particularly of nitrogen and minerals, reduces the osmotic load and the amount of undigested substrate entering the hindgut for microbial fermentation, both of which are key drivers of water influx into the intestines and subsequent wet litter (Angel et al., 2002).

In the present study, xylanase supplementation to WB and RB diet, increased villus height of duodenum and jejunum of broiler chickens. This enhancement in gut morphology is a key indicator of improved intestinal health and function, likely driven by a multi-factorial mechanism involving reduced anti-nutritional effects and enhanced nutrient supply to the enterocytes (Bedford et al., 2024). In this respect, Wu et al. (2004), reported increased ileal villus height by xylanase supplementation into the diet containing whole wheat. Villus alterations, especially making them taller, is often linked to better digestion and absorption of nutrients (Mathlouthi et al., 2002). Xylanase supplementation into diet containing insoluble fiber especially WB increase villus height in the duodenum by reducing viscosity, liberating nutrients, and promoting beneficial bacterial fermentation that produces SCFAs. This process stimulates enterocyte proliferation and improves gut health, ultimately leading to a larger absorptive surface area (Wu et al., 2004). Specifically, butyrate, a major SCFA produced from fiber fermentation, serves as a primary energy source for colonocytes and stimulates villus development through its role in cellular proliferation and differentiation (Topping and Clifton, 2001). Ceylan et al. (2024) indicated that several sources of 1,4-beta-xylanase decreased crypt depth in broiler chickens fed a corn-based diet, consistent with the results of the present investigation. Abdollahi et al. (2021) investigated the impact of different fiber sources and xylanase supplementation on egg production and gut shape. Xylanase supplementation independently or in combination with insoluble fiber (WB or sugar beet pulp), decreased crypt depth, indicating a similar trend with the present findings. The combined effect of increased villus height and decreased crypt depth results in a higher villus height to crypt depth ratio, which is a robust morphometric indicator of improved gut health, reflecting enhanced nutrient absorption and reduced tissue turnover (Angel et al., 2002). While Wu et al. (2004), observed that xylanase supplementation did not influence the number of goblet cells that is in contrast with the results of the present study. In a different study, Wang et al. (2024) discovered that adding xylanase at doses of 100 mg/kg and 150 mg/kg increased the number of goblet cells in the jejunum that is consistent with the results of the present investigation. Saki and Rahmatnezhad (2016), discovered that both soluble and insoluble dietary fibers augmented goblet cell populations in the ileum, corroborating the present findings. The increase in goblet cells observed in our study may be a direct response to the physicochemical changes in the gut environment, such as the presence of xylo-oligosaccharides (XOS), which can act as signaling molecules to stimulate mucin production as a protective mechanism (Ricke, 2018). Elevated soluble dietary fiber has been correlated with an increased quantity of goblet cells (Pourazadi et al., 2020b). Nonetheless, an increase in goblet cells signifies a thicker mucosal barrier that may hinder nutritional absorption (Pourazadi et al., 2020b). Whereas a decrease in goblet cell quantity might result in diminished mucin synthesis and reduced endogenous protein loss (Jacobs, 1983). This presents a paradox; however, it is likely that an optimal, rather than maximal, mucin layer is the goal. The xylanase-induced increase may represent a normalization of mucin output that was previously impaired by the inflammatory or abrasive nature of untreated fiber, thereby improving gut barrier function without excessively hindering absorption (Lillehoj et al., 2018). Furthermore, Wang et al. (2024) highlighted that xylanase enhances growth performance by regulating metabolism and improving gut health, supporting the hypothesis that a greater villus height to crypt depth ratio correlates with enhanced digestive efficacy and superior intestinal health (Adibmoradi et al., 2006; Hampson, 1986). Nonetheless, the principal reason for different findings between studies probably due to differences in the amounts of soluble and insoluble non-starch polysaccharides, as well as the particular xylanase source used. The specificity, side activities, and dosage of the xylanase product are critical factors, as different enzymes will generate unique profiles of oligosaccharides with varying prebiotic and stimbiotic effects, leading to divergent physiological outcomes (Biely et al., 2016). Moreover, dietary fiber can physically and enzymatically trigger numerous physiological processes, possibly modifying the histological characteristics of the gut. The initial health status of the gut and the existing microbial ecosystem are also pivotal, as they determine the response to enzymatic intervention and the subsequent fermentation of released oligosaccharides, ultimately shaping the morphological adaptation of the intestinal epithelium (Kers et al., 2018).

## Conclusion

The results of this study showed that xylanase supplementation to a diet containing WB or RB and also CTL reduced FCR and ileal viscosity, although this effect was greater in a diet containing WB. Also, adding the enzyme to a diet containing WB and RB increased the lactobacillus bacteria of the cecum. However, this study has some limitations. The experiment was conducted under controlled experimental conditions and with a specific broiler strain, which may not fully represent commercial production systems. Therefore, further studies are needed to validate these findings under commercial conditions and to explore the broader impacts of xylanase supplementation.

## CRediT authorship contribution statement

**Masoud Karimipour:** Investigation, Formal analysis. **Somayyeh Salari:** Writing – original draft, Supervision. **Ali Aghaei:** Writing – review & editing, Methodology.

## Disclosures

Authors have NO affiliations with or involvement in any organization or entity with any financial interest (such as honoraria; educational grants; participation in speakers’ bureaus; membership, employment, consultancies, stock ownership, or other equity interest; and expert testimony or patent-licensing arrangements), or non-financial interest (such as personal or professional relationships, affiliations, knowledge or beliefs) in the subject matter or materials discussed in this manuscript.
